# Find and treat or find and lose? Tuberculosis treatment outcomes among screened newly arrived asylum seekers in Germany 2002 to 2014

**DOI:** 10.2807/1560-7917.ES.2018.23.11.17-00042

**Published:** 2018-03-15

**Authors:** Anna Kuehne, Barbara Hauer, Bonita Brodhun, Walter Haas, Lena Fiebig

**Affiliations:** 1 Robert Koch Institute, Department for Infectious Disease Epidemiology, Respiratory Infections Unit, Berlin, Germany; 2 Postgraduate Training for Applied Epidemiology, Robert Koch Institute, Berlin, Germany affiliated to the European Programme for Intervention Epidemiology Training, ECDC, Stockholm, Sweden

**Keywords:** tuberculosis, refugees, international migration, Germany, mass screening, treatment outcome, public health surveillance

## Abstract

Germany has a low tuberculosis (TB) incidence. A relevant and increasing proportion of TB cases is diagnosed among asylum seekers upon screening. **Aim:** We aimed to assess whether cases identified by screening asylum seekers had equally successful and completely reported treatment outcomes as cases diagnosed by passive case finding and contact tracing in the general population. **Methods:** We analysed characteristics and treatment outcomes of pulmonary TB cases notified in Germany between 2002 and 2014, stratified by mode of case finding. We performed three multivariable analyses with different dependent variables: Model A: successful vs all other outcomes, Model B: successful vs documented non-successful clinical outcome and Model C: known outcome vs lost to follow-up. **Results:** TB treatment success was highest among cases identified by contact tracing (87%; 3,139/3,591), followed by passive case finding (74%; 28,804/39,019) and by screening asylum seekers (60%; 884/1,474). Cases identified by screening asylum seekers had 2.4 times higher odds of not having a successful treatment outcome as opposed to all other outcomes (A), 1.4 times higher odds of not having a successful treatment outcome as opposed to known non-successful outcomes (B) and 2.3 times higher odds of loss to follow-up (C) than cases identified by passive case finding. **Conclusion:** Screened asylum seekers had poorer treatment outcomes and were more often lost to follow-up. Linking patients to treatment facilities and investigating potential barriers to treatment completion are needed to secure screening benefits for asylum seekers and communities.

## Introduction

With 10.4 million new cases of active tuberculosis (TB) in 2016, TB remains one of the world’s biggest health threats [1]. Most countries in the European Union (EU) are low-incidence countries where TB predominantly affects vulnerable populations such as migrants, prisoners and people living with HIV [2]. To achieve an ongoing decrease in TB incidence in EU countries, further efforts are needed to address these often hard-to-reach groups [2]. In Germany, 5,915 cases of active TB were notified in 2016 [3]. Demographic changes and migration influence TB incidence in Germany and contributed to the end of a previously declining TB trend [3,4]. Ensuring early detection and comprehensive access of all population groups to timely and complete treatment will be essential to control TB and ultimately meet the World Health Organization’s (WHO) TB elimination goals [5].

Cases found by passive case finding, i.e. TB patients diagnosed after clinical presentation with symptoms or post mortem, contributed the highest proportion of new cases in 2016 (66%) [3]. Sixteen per cent of cases had been diagnosed by active case finding among asylum seekers and refugees [3]. This proportion was on average 2.4% between 2002 and 2014 and had been increasing since 2008, when it was smallest (0.7%) [3]. Active case finding is performed among several risk groups to ensure early detection and treatment and to prevent further transmission from infectious cases. In recently exposed persons, contact tracing is performed according to German contact tracing recommendations [6]. Among asylum seekers, screening is performed to find infectious pulmonary TB cases early at admission to shared accommodations (reception centres) after entering the country. Screening for infectious pulmonary TB at entry to such shared accommodations is mandatory according to §36.4 of the Protection Against Infection Act (Infektionsschutzgesetz (IfSG)). With the increasing number of migrants seeking asylum in Germany, the mandatory screening for infectious pulmonary TB among asylum seekers has challenged local public health authorities (LPHA) in 2014 and 2015 [7-11]. 

TB diagnosis – upon screening or clinical presentation – needs to be followed by rapid initiation of an effective and complete treatment to prevent further transmission, achieve cure and prevent the development of secondary drug resistance [12]. Tuberculosis treatment outcome monitoring is an essential part of TB surveillance and key for evaluating the effectiveness of TB screening and care. In line with international requirements [1,2], the German TB notification system comprises the treatment outcome categories *cured, treatment completed, died, treatment failure, treatment default, still on treatment, transfer out, missing* and *unable to determine* (Table 1). Treatment outcome is measured after 12 months follow-up and after 24 months for multidrug-resistant TB (MDR-TB) cases. The WHO and the Stop TB Partnership set the target of 90% treatment success (i.e. cured and treatment completed) for all TB cases that require treatment [1,13].

**Table 1:** Tuberculosis treatment outcome categories in the national notification system, Germany, 2002–2014

Categories	Definitions
Cured	Treatment completed and culture-negative samples taken at the end of the full course treatment and on at least one previous occasion
Treatment completed	Treatment completed without evidence of failure but no tests were performed or no result was available at the end of the full course of treatment
Died	Death before cure or treatment completion, irrespective of cause
Treatment failure	Culture or sputum smear remaining positive or becoming positive again 5 months or more into the course of treatment
Treatment default^a^	Treatment interrupted for at least 2 consecutive months
Still on treatment	Patient still on treatment at 12 months (and at 24 months for multidrug-resistant (MDR) TB cases) without any other outcome during treatment
Transfer out	Patient referred to a known or unknown address and information on outcome not available
Missing	Information on treatment outcome is missing (empty field)
Unable to determine	Information on treatment outcome could not be obtained by the local public health authority

**^a^**‘Treatment default’ is the translation of the German category name ‘Abbruch der Behandlung’. Internationally, the previously used term ‘defaulter’ has recently been replaced by ‘lost to follow-up’, but this is not reflected in the German TB surveillance system.

To what extent pulmonary TB found among screened asylum seekers in Germany is followed up until treatment completion, remains unclear however. We therefore aimed to assess whether TB cases identified by screening among asylum seekers had an equally successful and completely reported treatment outcome as those diagnosed by passive case finding and by contact tracing, in order to highlight potential gaps in surveillance and case management.

## Methods

### Data source

We used case-based national TB notification data from Germany, reported to the Robert Koch Institute (RKI) through the electronic reporting system SurvNet@RKI [14]. Date of data extraction was 1 March 2016. 

We included in our analysis pulmonary TB cases notified between 2002 and 2014 with available information on age and sex (total n = 52,995). The dataset was further restricted to cases that were identified by the following modes of case finding: (i) screening of asylum seekers, (ii) passive case finding and (iii) contact tracing (total n = 44,084). The notification system, case definitions for TB and diagnostic procedures have remained largely unchanged over the investigation period.

### Definitions

For German national disease surveillance, a case of TB is defined by clinical diagnosis of TB by a physician followed by the decision to initiate a full course of anti-tuberculosis treatment, with or without bacteriological confirmation or epidemiological link [15]. Bacteriological confirmation refers to a culture of *Mycobacterium tuberculosis* complex, or a combination of a positive microscopy result for acid-fast bacilli with a positive nucleic acid amplification test (NAAT) for the same specimen type [15]. In the TB notification system, information on the patients’ age, sex, country of birth and the mode of case finding is recorded, as is bacteriological testing including drug resistance, previous TB diagnosis, site of TB and treatment outcome at any time of follow-up, in this study set to at least 12 months [16].

Modes of case finding are defined by reporting guidelines [16]. Screened asylum seekers are defined as TB cases that were identified by screening asylum seekers according to §36.4 IfSG by chest X-ray (except pregnant women or children younger than 15 years) on admission to a shared accommodation [17]. In children and pregnant women, screening including clinical signs and symptoms and immunological testing with either interferon-γ release assay (IGRA) or tuberculin skin test (TST) is recommended [17,18]. Thus, the screening intended to rule out infectious pulmonary TB also leads to the detection of pulmonary TB with negative bacteriological result. Cases identified by passive case finding are defined as TB cases diagnosed after clinical presentation; TB cases diagnosed post mortem were excluded from our study. Cases identified during the follow-up of exposed persons are defined as cases identified by contact tracing. Cases identified by other active case finding were not considered in this study.

### Treatment success and data completeness

**Figure 1:**
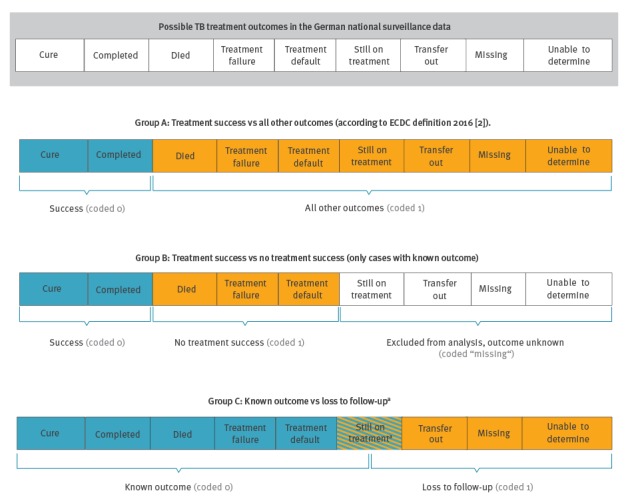
Grouping and coding of treatment outcomes of notified tuberculosis cases in the national notification system, Germany, 2002–2014

ECDC: European Centre for Disease Prevention and Control; TB: tuberculosis.

^a^ Still on treatment is regarded as loss to follow-up when disease onset is more than 24 months ago for non-MDR-TB or more than 36 months ago for MDR-TB, the remaining cases are regarded as cases with known outcome.

To assess treatment success and data completeness, we grouped treatment outcomes in three different ways (Group A, B and C) as displayed in Figure 1.

For this study, ‘loss to follow-up’ describes cases that were ‘lost’ to the national TB notification system, i.e. the outcome cannot be evaluated. This should be distinguished from the newly introduced term ‘lost to follow-up’ that has replaced ‘defaulter’ in international TB reports and describes a known treatment interruption for at least two consecutive months.

**Group A**: Cases that were *cured* or had *treatment completed* were referred to as cases with *successful treatment* and compared with cases that were recorded with all other outcomes. This definition is adapted from ECDC classification 2016 [2] and is in line with WHO and the Stop TB Partnership’s definitions of treatment outcomes [1,13].

**Group B**: Cases with successful treatment were compared with cases with known non-successful treatment outcomes (*died*, *treatment failure* or *default);* all cases with outcome categories that contained essentially no information on the result of the treatment of the case (*still on treatment, transfer out, missing* and *unable to determine)* were excluded from this comparison in order to disentangle cases with non-successful treatment outcome from cases that were *lost to follow-up*.

**Group C**: Cases with known treatment outcomes, both successful and unsuccessful *(successful treatment, died, treatment failure, treatment default)* were compared with cases that were *lost to follow-up* to the national tuberculosis notification system (*transfer out, missing, unable to determine* or too long *still on treatment*). The classification is based on the assumption that in all these cases, LPHA did presumably not have up-to-date information and could not ascertain the treatment outcome. ‘Too long’ *still on treatment* was defined as cases without MDR-TB who were notified as *still on treatment* more than 24 months after notification and MDR-TB cases who were notified as *still on treatment* more than 36 months after notification. The remaining cases notified as *still on treatment* were defined as cases with known outcome as still on treatment is valid information on the treatment status.

### Data analysis and protection

We describe demographic information, i.e. age (continuous), sex (female vs male), country of birth (Germany vs WHO regions excluding Germany vs unknown), as well as clinical information, i.e. MDR (not applicable, no drug susceptibility test (DST) reported, DST reported and among those with DST: not MDR vs MDR), previous TB diagnosis (no vs yes vs unknown), infectiousness (respiratory specimen: culture-negative and smear-negative vs culture-positive and smear-negative vs smear-positive vs unknown), severity of disease (pulmonary TB with TB of the central nervous system (CNS), meningitis or disseminated TB vs pulmonary TB only or with other secondary sites vs pulmonary TB with unknown additional manifestations) and treatment outcomes (Figure 1) by mode of case finding.

We also describe the above characteristics by treatment outcome. For categorical variables, we present numbers and proportions, for continuous variables, median and interquartile range (IQR).

The associations between mode of case finding and treatment outcome or loss to follow-up were investigated with multivariable logistic regression analyses using the passive case finding group as reference group. We interpreted coefficients in terms of odds ratios (OR) and report 95% confidence intervals (CI). We designed three logistic regression models (A, B, C) with different dependent variables: one for each group (A, B, C) of treatment outcome and loss to follow-up (Figure 1). We included mode of case finding as the independent variable and the following potential confounders (as described above if not specified) in all three models: age (in groups of 15 years), sex, country of birth (simplified: Germany vs other vs unknown), drug resistance (simplified: not MDR, MDR, unknown), infectiousness, previous TB and severity of disease, as well as reporting period (2002–05 vs 2006–14) as changes in data plausibility checks and completeness checks were introduced in 2006.

Analyses were conducted with STATA version 14 (Stata corporation, Texas, United States).

All investigated data were anonymous and collected within the legal framework of the IfSG.

## Results

The cases’ demographic and clinical characteristics stratified by mode of case finding are presented in Table 2.

**Table 2:** Demographic and clinical characteristics of pulmonary tuberculosis cases notified by mode of case finding, Germany, 2002–2014 (n = 44,084)

Characteristics of cases		Active case finding				Passive case finding	
		Screening asylum seekers		Contact tracing		Diagnosis subsequent to clinical presentation	
		N = 1,474		N = 3,591		N = 39,019	
		n	% of N	N	% of N	n	% of N
**Demographic characteristics **							
Median age in years (IQR)		28	(22-37)	27	(11-44)	50	(34-68)
Sex							
Female		336	23	1,628	45	14,373	37
Male		1,138	77	1,963	55	24,646	63
Place of birth							
Germany		6	0.4	2,312	64	21,420	55
Other country	WHO Region Europe without Germany	539	37	773	21	10,156	26
	WHO Region Eastern Mediterranean	377	26	104	2.9	1,682	4.3
	WHO Region Africa	320	22	84	2.3	1,722	4.4
	WHO Region South-East Asia	46	3.1	74	2.1	1,345	3.4
	WHO Region Western Pacific	82	5.6	58	1.6	997	2.6
	WHO Region Americas	4	0.3	11	0.3	266	0.7
Unknown country		100	6.8	175	4.9	1,431	3.7
**Clinical characteristics **							
Infectiousness							
Culture-negative, smear-negative		496	34	1,164	32	6,268	16
Culture-positive, smear-negative		498	34	1,417	39	12,737	33
Smear-positive		410	28	748	21	18,966	49
Unknown		70	4.7	262	7.3	1,048	2.7
Previous TB							
No		876	59	3,252	91	29,963	77
Yes		201	14	109	3.0	4,429	11
Unknown		397	27	230	6.4	4,627	12
Drug resistance							
Drug susceptibility test (DST reported)		838	57	2,037	57	28,950	74
Not MDR (% of DST reported)		747	89	2,004	98	28,388	98
MDR (% of DST reported)		91	11	33	1.6	562	1.9
Unknown	Not applicable; bacteriologically negative	486	33	1,129	31	6,075	16
	No drug susceptibility test reported	150	10	425	12	3,994	10
Severity of disease							
Exclusively pulmonary TB		1,173	80	3,081	86	32,620	84
Pulmonary and CNS, meningitis or disseminated TB		5	0.3	10	0.3	574	1.5
Unknown		296	20	500	15	5,825	15

CNS: central nervous system; DST: drug susceptibility test; MDR: multidrug-resistant; TB: tuberculosis; WHO: World Health Organization.

Cases identified by screening asylum seekers (n = 1,474) were of similar median age with a smaller IQR compared with cases identified by contact tracing (n = 3,591) (28 vs 27 years) and were less often female (23% vs 45%). They had a similar proportion of culture- and smear-negative cases (34% vs 32%), more often unknown information about previous TB (27% vs 6.4%) and more often MDR-TB (11% vs 1.6%) (Table 2). Compared with cases identified by passive case finding (n = 39,019), the cases identified by screening asylum seekers had a lower median age (28 vs 50 years), a lower proportion of females (23% vs 37%) and a higher proportion of culture- and smear-negative cases (34% vs 16%), of unknown information about previous TB (27% vs 12%) and of MDR-TB (11% vs 1.9%) (Table 2).

**Figure 2:**
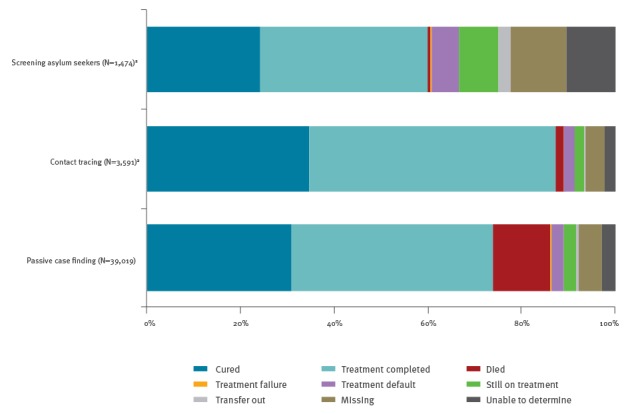
Treatment outcomes of notified pulmonary tuberculosis cases by mode of case finding, Germany, 2002–2014 (n = 44,084)

^a^ Active case finding.

Treatment success was highest among pulmonary TB cases identified by contact tracing (87%; 3,139/3,591), followed by cases identified by passive case finding (74%; 28,804/39,019) and by screening asylum seekers (60%; 884/1,474) (Figure 2).

The largest proportion of missing and indeterminate data on treatment outcome was among cases identified by screening asylum seekers (22%; 329/1,474), followed by patients identified by passive case finding (7.9%; 3,076/39,019) and contact tracing (6.3%; 225/3,591) (Figure 2).

Detailed analyses showed that the proportion of successful outcomes among all outcomes (Group A, Figure 1) varied not only by the mode of case finding but also by age, sex, place of birth, infectiousness, previous TB diagnosis treatment, drug resistance, severity of disease and changes in data plausibility and completeness checks (Table 3).

**Table 3:** Demographic and clinical characteristics of notified pulmonary tuberculosis cases by treatment outcome, Germany, 2002–2014 (n = 44,084)

Characteristics of cases		Group A: successful outcomes (n) among all outcomes (N)			Group B: successful outcomes (n) among known outcomes (N)			Group C: known outcomes (n) among all outcomes (N)		
		n	N	% of N	n	N	% of N	n	N	% of N
**Main exposure of interest**										
Mode of case finding										
Passive case finding		28,804	39,019	74	28,804	34,766	83	35,214	39,019	90
Contact tracing		3,139	3,591	87	3,139	3,285	96	3,334	3,591	93
Screening asylum seekers		884	1,474	60	884	983	90	1,062	1,474	72
**Demographic characteristics**										
Age in years										
< 15		1,611	1,782	90	1,611	1,634	99	1,654	1,782	93
15–29		6,329	7,874	80	6,329	6,645	95	6,777	7,874	86
30–44		8,256	10,322	80	8,256	8,959	92	9,105	10,322	88
45–59		7,536	9,674	78	7,536	8,652	87	8,769	9,674	91
60–74		5,649	8,070	70	5,649	7,342	77	7,432	8,070	92
≥ 75		3,446	6,362	54	3,446	5,802	59	5,873	6,362	92
Sex										
Female		12,699	16,337	78	12,699	14,557	87	14,761	16,337	90
Male		20,128	27,747	73	20,128	24,477	82	24,849	27,747	90
Place of birth										
Germany		17,337	23,738	73	17,337	21,653	80	21,950	23,738	92
Other country	WHO Region Europe	8,850	11,468	77	8,850	10,077	88	10,192	11,468	89
	WHO Region Eastern Mediterranean	1,701	2,163	79	1,701	1,848	92	1,892	2,163	87
	WHO Region Africa	1,675	2,126	79	1,675	1,794	93	1,819	2,126	86
	WHO Region South-East Asia	1,141	1,465	78	1,141	1,233	93	1,252	1,465	85
	WHO Region Western Pacific	886	1,137	78	886	955	93	970	1,137	85
	WHO Region Americas	232	281	83	232	244	95	248	281	88
Unknown country		1,005	1,706	59	1,005	1,230	82	1,287	1,706	75
**Clinical characteristics**										
Infectiousness										
Culture-negative, smear-negative		6,128	7,928	77	6,128	7,118	86	7,288	7,928	92
Culture-positive, smear-negative		11,035	14,652	75	11,035	13,089	84	13,212	14,652	90
Smear-positive		14,806	20,124	74	14,806	17,750	83	17,984	20,124	89
Unknown		858	1,380	62	858	1,077	80	1,126	1,380	82
Previous TB										
No		26,433	34,091	77	26,433	30,690	86	31,090	34,091	91
Yes		3,188	4,739	67	3,188	4,132	77	4,193	4,739	88
Unknown		3,206	5,254	61	3,206	4,212	76	4,327	5,254	82
Drug resistance										
Not MDR		24,034	31,139	77	24,034	28,066	86	28,214	31,139	91
MDR		399	686	58	399	505	79	536	686	78
Unknown	Not applicable; bacteriologically negative	5,963	7,690	77	5,963	6,930	86	7,089	7,690	92
	No drug susceptibility test reported	2,431	4,569	53	2,431	3,533	69	3,762	4,569	82
Severity of disease										
Exclusively pulmonary TB		28,163	36,874	76	28,163	33,114	85	33,583	36,874	91
Pulmonary and CNS, meningitis or disseminated TB		342	589	58	342	520	66	527	589	89
Unknown		4,322	6,621	65	4,322	5,400	80	5,500	6,621	83
**Time period based on change of data plausibility and completeness checks**										
2002–05		12,594	17,310	73	12,594	14,978	84	15,251	17,310	88
2006–14		20,233	26,774	76	20,233	24,056	84	24,359	26,774	91

CNS: central nervous system; DST: drug susceptibility test; MDR: multidrug-resistant; TB: tuberculosis; WHO: World Health Organization.

Treatment success (Group A) was particularly low among TB cases who had no DST reported (53%; 2,431/4,569), were 75 years or older (54%; 3,446/6,362), had MDR-TB (58%; 399/686), a severe TB manifestation (CNS, meningitis or disseminated) in addition to pulmonary TB (58%; 342/589), unknown place of birth (59%; 1,005/1,706) or were identified by screening asylum seekers (60%; 884/1,474) (Table 3).

Analysis adjusted for demographic and clinical characteristics showed that mode of case finding was independently associated with treatment success (Model A, Figure 1, Table 4). It indicated 2.4 times higher odds of non-successful treatment for cases identified by screening asylum seekers compared with cases identified by passive case finding; cases identified by contact tracing showed 0.64 times lower odds of non-successful treatment outcomes compared with passive case finding (Table 4).

**Table 4:** Notified pulmonary tuberculosis cases - multivariable analyses for the association between the mode of case finding and treatment outcome, Germany, 2002–2014 (n = 44,084)

Characteristics of cases	Model A: success (0) vs all other outcomes (1)			Model B: success (0) vs no treatment success (1)			Model C: known outcome (0) vs loss to follow-up (1)		
	aOR	95% CI	p value	aOR	95% CI	p value	aOR	95% CI	p value
**Main exposure of interest **									
Mode of case finding									
Passive case finding	Ref			Ref			Ref		
Contact tracing	0.64	0.57–0.71	< 0.001	0.54	0.45–0.65	< 0.001	0.73	0.63–0.84	< 0.001
Screening asylum seekers	2.37	2.11–2.67	< 0.001	1.38	1.10–1.73	0.006	2.35	2.06–2.68	< 0.001
**Demographic characteristics **									
Age in years									
< 15	Ref			Ref			Ref		
15–29	1.84	1.54–2.20	< 0.001	3.06	1.98–4.73	< 0.001	1.41	1.15–1.74	< 0.001
30–44	1.90	1.59–2.26	< 0.001	4.79	3.13–7.34	< 0.001	1.25	1.02–1.54	0.030
45–59	2.11	1.77–2.51	< 0.001	7.43	4.86–11.35	< 0.001	1.06	0.86–1.30	0.571
60–74	3.09	2.59–3.68	< 0.001	14.53	9.52–22.19	< 0.001	0.84	0.68–1.04	0.116
≥ 75	6.39	5.35–7.63	< 0.001	34.15	22.36–52.16	< 0.001	0.82	0.66–1.02	0.081
Sex									
Female	Ref			Ref			Ref		
Male	1.33	1.26–1.39	< 0.001	1.52	1.42–1.62	< 0.001	1.07	1.00–1.14	0.049
Place of birth									
Germany	Ref			Ref			Ref		
Other country	0.90	0.85–0.94	< 0.001	0.70	0.65–0.75	< 0.001	1.34	1.25–1.45	< 0.001
Unknown country	1.81	1.61–2.03	< 0.001	0.94	0.80–1.12	0.494	3.10	2.72–3.53	< 0.001
**Clinical characteristics**									
Infectiousness									
Culture-negative, smear-negative	Ref			Ref			Ref		
Culture-positive, smear-negative	2.58	2.35–2.84	< 0.001	2.35	2.08–2.65	< 0.001	2.19	1.91–2.50	< 0.001
Smear-positive	2.79	2.55–3.05	< 0.001	2.54	2.27–2.85	< 0.001	2.31	2.04–2.62	< 0.001
Unknown	2.12	1.86–2.41	< 0.001	1.64	1.37–1.97	< 0.001	2.43	2.06–2.87	< 0.001
Previous TB treatment									
No	Ref			Ref			Ref		
Yes	1.20	1.12–1.29	< 0.001	1.18	1.08–1.28	< 0.001	1.23	1.11–1.36	0.003
Unknown	1.90	1.78–2.03	< 0.001	1.84	1.69–1.97	< 0.001	1.73	1.59–1.88	< 0.001
Drug resistance									
No MDR	Ref			Ref			Ref		
MDR	2.83	2.40–3.32	< 0.001	3.03	2.40–3.83	< 0.001	1.89	1.56–2.30	< 0.001
Unknown	2.76	2.56–2.98	< 0.001	2.56	2.33–2.81	< 0.001	1.83	1.65–2.02	< 0.001
Severity of disease									
Exclusively pulmonary TB	Ref			Ref			Ref		
Pulmonary and CNS, meningitis or disseminated TB	2.51	2.10–2.99	< 0.001	3.25	2.63–4.00	< 0.001	1.23	0.94–1.62	0.125
Unknown	1.48	1.40–1.58	< 0.001	1.24	1.15–1.35	< 0.001	1.83	1.70–1.98	< 0.001
**Time period based on change of data plausibility and completeness checks **									
2002–05	Ref			Ref			Ref		
2006–14	0.91	0.86–0.95	< 0.001	1.04	0.98–1.10	0.228	0.75	0.70–0.80	< 0.001

aOR: adjusted odds ratio; CI: confidence interval; CNS: central nervous system; MDR: multidrug-resistant; TB: tuberculosis.

Restricting analysis of treatment outcomes to cases with known outcomes (Group B, Figure 1) and comparing successful and non-successful treatment among them, treatment success was particularly low for cases  aged 75 years or older (59%; 3,446/5,802), with severe manifestation in addition to pulmonary TB (66%; 342/520) and cases that had no DST reported (69%; 2,431/3,533) (Table 3). While cases identified by screening asylum seekers had higher treatment success (90%; 884/983) than cases identified by passive case finding (83%; 28,804/34,766) in the descriptive analysis of Group B, adjusted analysis indicated 1.4 times higher odds for a non-successful treatment outcome for cases identified by screening asylum seekers compared with cases identified by passive case finding (Model B, Table 4). In addition, older age, MDR-TB, severe manifestations and infectiousness were associated with particularly high odds of unsuccessful treatment outcome in Model B (Table 4).

Analysis of loss to follow-up (Group C) showed that the proportion of known outcomes among all possible treatment outcomes was lowest among cases identified by screening asylum seekers (72%; 1,062/1,474), followed by cases with unknown place of birth (75%; 1,287/1,706) and cases with MDR (78%; 536/686) (Table 3). Adjusted analysis indicated 2.3 times higher odds for loss to follow-up among cases identified by screening asylum seekers compared with cases identified by passive case finding; cases identified by contact tracing showed 27% lower odds of loss to follow-up compared with passive case finding (Model C, Table 4). Apart from identification by screening asylum seekers, cases with unknown place of birth and unknown drug resistance showed particularly high odds of getting lost to follow-up (Table 4).

## Discussion

With our study, we aimed to assess treatment outcomes of pulmonary TB cases identified by screening asylum seekers. We found that cases identified by screening asylum seekers – unlike cases identified by contact tracing – had significantly poorer treatment outcomes and higher odds of loss to follow-up compared with cases identified by passive case finding after adjustment for demographic and clinical characteristics.

Cases identified by screening asylum seekers were similar to those identified by contact tracing in terms of age, infectiousness and severity of disease but were more likely to have MDR-TB or a history of previous TB diagnosis. Compared with cases identified by passive case finding, those among screened asylum seekers were younger, more often male and less infectious.

Our study results corroborate previous findings that TB screening by chest X-ray, as used for asylum seekers, allowed early detection of cases that were still smear-negative [19-22]. Thus screening has the potential to prevent transmission and facilitate early treatment initiation. The high proportion of bacteriologically negative pulmonary TB cases (34%) raises the issue of potential overdiagnosis of TB by chest X-ray screening. However, TB cases identified by contact tracing were equally often bacteriologically negative, and the detection of bacteriologically negative TB is considered beneficial given the high risk of developing infectious TB if untreated [23]. In addition, screened asylum seekers were more likely to have had a diagnosis of MDR-TB or a positive or unknown history of previous TB compared with cases identified by passive case finding. Contributing factors may include higher rates of TB and MDR-TB in their country of birth [1] and fragmented healthcare services in countries of origin leading to treatment interruptions [24]. Of note, DST results, which are strongly warranted for treatment decisions, were frequently unavailable.

With 60% treatment success (Group A) among pulmonary TB cases identified by screening asylum seekers, 74% among those identified by passive case finding and 87% among those identified by contact tracing, none of the groups met the target of 90% treatment success [1,13]. However, the odds for non-successful treatment were substantially higher for cases identified by screening asylum seekers and markedly lower for cases identified by contact tracing compared to the cases identified by passive case finding (adjusted for other known confounders).

While asylum seeker status was independently associated with an unsuccessful treatment outcome, this was not true for reporting a foreign country of birth, which had lower odds of non-successful treatment outcomes (Model A). Other studies also found poorer treatment adherence for migrants with insecure legal status [25] and those that had arrived recently [26], but did not find an independent negative impact of foreign country of origin. Our findings contrast with a recent analysis of European data that found poorer treatment outcomes for foreign-born cases; however, that study could not distinguish between legal status and country of birth [27].

The magnitude of association between known clinical risk factors and non-successful treatment outcomes was greater when unknown outcomes were excluded from the analysis (Model B). In accordance with previous knowledge [20,27,28], increasing age, a proxy for co-morbidities and risk of dying, strongly increased the odds of non-successful treatment in model B. In addition, the odds for MDR-TB and severe disease manifestations were greater among those with negative treatment outcomes in model B compared with model A, in coherence with previous studies [20,29-32]. 

In our study, being identified by screening asylum seekers was also independently associated with being lost to follow-up after adjustment for potential confounders. Reasons for loss to follow-up can include the patient’s decision to stop treatment without informing treatment facilities [33]. A potential explanation for this may be the lack of perceived illness [34] that might be more pronounced in cases identified by screening who had no symptoms. That patients identified by contact tracing were 27% less likely to be lost to follow-up than patients identified by passive case finding, however, indicates that even asymptomatic patients can be successfully followed up. Geographical distance to TB-treatment facilities, a trusting and supportive provider–patient relationship as well as security of legal status have been found to be predictors for treatment adherence [25,34-36]. The reduction of structural barriers to TB diagnosis and treatment including availability of free, accessible and culturally appropriate health services for vulnerable groups such as migrants has been shown to be a key element in increasing treatment success [36]. Potential structural barriers to TB treatment completion and reasons for loss to follow-up in Germany include limited access to care and interpreters [7], (forced) relocations of asylum seekers within Germany or to other countries during treatment [7,11,33] and changing administrative authorities handling the case [10]. 

### Limitations

Our investigation was based on national notification data, and under- or overestimation of the true case number owing to under-diagnosis and under-reporting or to double-reporting cannot be entirely excluded. In addition, incomplete information for notified cases at the national level may reflect unavailable data at the treatment facility or at the LPHA level. Non-reported treatment outcome cannot entirely be disentangled from non-completed treatment in our data. The TB patient may have completed treatment within the remit of a different health authority than the one that received the initial notification, or under the supervision of a doctor or hospital that has missed to report the treatment outcome to the local health authority.

Based on the available variables in the notification data, our analysis could only compare ‘asylum seekers identified by screening’ with cases identified by other modes of case finding. However, cases identified by other modes of case finding may also be asylum seekers. Furthermore, incorrect classification of the mode of case finding cannot be excluded.

Full information on tuberculosis treatment outcome becomes available only 1 year after the reporting year and our study therefore includes cases notified up to and including 2014. Whether characteristics and treatment outcomes of cases notified from 2002 to 2014 can be extrapolated to cases notified later is unknown and will require evaluation. Increasing workload at LPHA level caused by increasing case numbers (by nearly 30% in 2015 [3]) may affect the follow-up of cases and completion of information.

## Conclusion 

The low proportion of smear-positive TB suggests that asylum seekers were found early by screening; a good starting point for successful treatment. However, they were often lost to follow-up and had poorer treatment outcomes than cases identified by passive case finding or contact tracing.

The documentation of mode of case finding in German TB notification data proved useful for the evaluation of group-specific treatment outcomes, namely screened asylum seekers. We recommend a standardised approach to reporting of case finding information across Europe to allow evaluation of treatment success and comparison across countries by modes of case finding. Regarding treatment outcome data, we need to better disentangle non-reported treatment outcomes from reporting incomplete course of treatment and to address both issues individually to obtain high treatment success. 

Increased case detection by screening can only unfold its health benefits when detected tuberculosis is effectively treated and reliably cured. Tuberculosis screening activities among asylum seekers can be a door to access general medical care. TB screening at admission to reception centres may also reduce TB exposure and reduce the need for resource-intensive contact investigations in these settings.

While specific reasons for the higher odds of non-successful treatment among asylum seekers in Germany need to be studied further, available research suggests that patients need to be better linked to treatment facilities and structural barriers to treatment completion need to be addressed to secure screening benefits for asylum seekers and the communities.

## References

[1] World Health Organization (WHO). Global tuberculosis report 2017. Geneva: WHO; 2017. Available from: http://apps.who.int/iris/bitstream/10665/259366/1/9789241565516-eng.pdf?ua=1

[2] European Centre for Disease Prevention and Control (ECDC)/WHO Regional Office for Europe. Tuberculosis surveillance and monitoring in Europe 2016. Stockholm: ECDC; 2016. Available from: https://ecdc.europa.eu/en/publications-data/tuberculosis-surveillance-and-monitoring-europe-2016

[3] Robert Koch Institute (RKI). Bericht zur Epidemiologie der Tuberkulose in Deutschland für 2016. [German tuberculosis surveillance report 2016]. Berlin: RKI; 2017. German. Available from: https://www.rki.de/DE/Content/InfAZ/T/Tuberkulose/Download/TB2016.pdf;jsessionid=A7701BC5522F7BB961DFA52EB767FFD1.1_cid381?__blob=publicationFile

[4] Fiebig L Hauer B Brodhun B Altmann D Haas W Tuberculosis in Germany: a declining trend coming to an end? Eur Respir J. 2016;47(2):667-70. 10.1183/13993003.01410-2015 26493803

[5] World Health Organization (WHO). The global plan to stop TB 2011-2015: Transforming the fight towards elimination of tuberculosis. Geneva: WHO; 2011. Available from: http://apps.who.int/iris/bitstream/10665/44437/1/9789241500340_eng.pdf

[6] Diel R Loytved G Nienhaus A Castell S Detjen A Geerdes-Fenge H Neue Empfehlungen für die Umgebungsuntersuchungen bei Tuberkulose. Deutsches Zentralkomitee zur Bekämpfung der Tuberkulose. [New recommendations for contact tracing in tuberculosis. German Central Committee against Tuberculosis]. Pneumologie. 2011;65(6):359-78. German. 10.1055/s-0030-1256439 21560113

[7] Robert Koch Institute (RKI) Untersuchung von Asylsuchenden und Flüchtlingen auf TB – Onlinebefragung des DZK. [Screening asylum seekers and refugees for tuberculosis – an online survey by the German Central Committee against Tuberculosis (DZK)]. Epid Bulletin. 2016;10/11:84-6. German.

[8] Halder G Zühl J (Tuberculosis among asylum seekers in Munich) Tuberkulose bei Asylbewerbern in München. J. Gesundheitswesen. 2016;78(4):V26.

[9] Robert Koch Institute (RKI) Tuberkulose-Screening im Rahmen der infektionshygienischen Untersuchung Asylbegehrender in Rheinland-Pfalz, Trier. [Tuberculosis screening as part of the entrance examination for asylum seekers in Rhineland –Palatinate, Trier]. Epid Bulletin. 2015;11/12:88-90. German.

[10] Schönfeld N. Tuberkulose bei Geflüchteten – was Sie beachten sollten. [Tuberculosis among refugees – what you should consider]. Pneumo News. 2016;8(7S):1-5. German. Available from: https://www.bgw-online.de/DE/Arbeitssicherheit-Gesundheitsschutz/Grundlagen-Forschung/GPR-Medientypen/Downloads/Tb-Fluechtlinge_Download.pdf?__blob=publicationFile

[11] Dreweck C, Kerner E, Gullich K, Halder G. Die soziale Dimension der Tuberkulose in der Stadt München. [The social dimension of tuberculosis in the city of Munich]. Gesundheitswesen. 2013;75(11):689-92. German. Available from: https://www.thieme-connect.com/products/ejournals/abstract/10.1055/s-0033-1357154 10.1055/s-0033-1357154 24285156

[12] Schaberg T Bauer T Castell S Dalhoff K Detjen A Diel R Empfehlungen zur Therapie, Chemoprävention und Chemoprophylaxe der Tuberkulose im Erwachsenen- und Kindesalter. Deutsches Zentralkomitee zur Bekämpfung der Tuberkulose (DZK), Deutsche Gesellschaft für Pneumologie und Beatmungsmedizin (DGP). [Recommendations for therapy, chemoprevention and chemoprophylaxis of tuberculosis in adults and children. German Central Committee against Tuberculosis (DZK), German Respiratory Society (DGP)]. Pneumologie. 2012;66(3):133-71. German. 22328186 10.1055/s-0031-1291619

[13] Stop TB. Partnership. The paradigm shift 2016-2020. Global plan to end TB. Geneva: Stop TB Partnership; UN OPS; 2015. Available from: http://www.stoptb.org/assets/documents/global/plan/GlobalPlanToEndTB_TheParadigmShift_2016-2020_StopTBPartnership.pdf

[14] Faensen D Claus H Benzler J Ammon A Pfoch T Breuer T SurvNet@RKI--a multistate electronic reporting system for communicable diseases. Euro Surveill. 2006;11(4):614 10.2807/esm.11.04.00614-en 16645245

[15] Robert Koch Institute (RKI). Falldefinitionen des Robert Koch-Instituts zur Übermittlung von Erkrankungs- oder Todesfällen und Nachweisen von Krankheitserregern. [Case definitions from the Robert Koch Institute for reporting of disease cases or deaths and of pathogens]. Berlin: RKI; 2015. German. Available from: https://www.rki.de/DE/Content/Infekt/IfSG/Falldefinition/Downloads/Falldefinitionen_des_RKI.pdf?__blob=publicationFile

[16] Robert Koch Institute (RKI). Leitfaden zur Übermittlung von Fallberichten zur Tuberkulose. [Surveillance reporting guidelines for case notifications of tuberculosis]. Berlin: RKI; 2004. German. Available from: http://www.rki.de/DE/Content/InfAZ/T/Tuberkulose/Download/TB_Leitfaden.pdf?__blob=publicationFile

[17] Robert Koch Institute (RKI). Thorax-Röntgenuntersuchungen bei Asylsuchenden gemäß § 36 Absatz 4 IfSG. [Chest X-ray examination among asylum seekers according to § 36.4 Protection Against Infection Act]. Berlin: RKI; 2015. German. Available from: http://www.rki.de/DE/Content/InfAZ/T/Tuberkulose/Tuberkulose_Roentgen-Untersuchungen_Asylsuchende.html

[18] Robert Koch Institute (RKI). Untersuchung auf Tuberkulose bei asylsuchenden Kindern und Jugendlichen < 15 Jahre. [Tuberculosis diagnostics among asylum seeking children and teens < 15 years of age]. Berlin: RKI; 2015. German. Available from: http://www.rki.de/DE/Content/InfAZ/T/Tuberkulose/Tuberkulose-Screening_Kinder.html

[19] Tsuda Y Matsumoto K Komukai J Furukawa K Saito K Shimouchi A Tuberculosis screening by chest radiography among international students at Japanese language schools in Osaka city. Kekkaku. 2015;90(10):677-82. Japanese. 26821397

[20] Story A Aldridge RW Abubakar I Stagg HR Lipman M Watson JM Active case finding for pulmonary tuberculosis using mobile digital chest radiography: an observational study. Int J Tuberc Lung Dis. 2012;16(11):1461-7. 10.5588/ijtld.11.0773 22981252

[21] Kranzer K Afnan-Holmes H Tomlin K Golub JE Shapiro AE Schaap A The benefits to communities and individuals of screening for active tuberculosis disease: a systematic review. Int J Tuberc Lung Dis. 2013;17(4):432-46. 10.5588/ijtld.12.0743 23485377

[22] Verver S Bwire R Borgdorff MW Screening for pulmonary tuberculosis among immigrants: estimated effect on severity of disease and duration of infectiousness. Int J Tuberc Lung Dis. 2001;5(5):419-25. 11336272

[23] Aldridge RW Zenner D White PJ Williamson EJ Muzyamba MC Dhavan P Tuberculosis in migrants moving from high-incidence to low-incidence countries: a population-based cohort study of 519 955 migrants screened before entry to England, Wales, and Northern Ireland. Lancet. 2016;388(10059):2510-8. 10.1016/S0140-6736(16)31008-X 27742165 PMC5121129

[24] Cousins S Experts sound alarm as Syrian crisis fuels spread of tuberculosis. BMJ. 2014;349(dec03 5):g7397. 10.1136/bmj.g7397 25471893

[25] Lin S Melendez-Torres GJ Systematic review of risk factors for nonadherence to TB treatment in immigrant populations. Trans R Soc Trop Med Hyg. 2016;110(5):268-80. 10.1093/trstmh/trw025 27198210 PMC4914875

[26] Cegolon L Maguire H Mastrangelo G Carless J Kruijshaar ME Verlander NQ Predictors of failure to complete tuberculosis treatment in London, 2003-2006. Int J Tuberc Lung Dis. 2010;14(11):1411-7. 20937180

[27] Karo B Hauer B Hollo V van der Werf MJ Fiebig L Haas W Tuberculosis treatment outcome in the European Union and European Economic Area: an analysis of surveillance data from 2002-2011. Euro Surveill. 2015;20(49):30087. 10.2807/1560-7917.ES.2015.20.49.30087 26676247

[28] Hauer B Brodhun B Altmann D Fiebig L Loddenkemper R Haas W Tuberculosis in the elderly in Germany. Eur Respir J. 2011;38(2):467-70. 10.1183/09031936.00199910 21804163

[29] Ditah IC Reacher M Palmer C Watson JM Innes J Kruijshaar ME Monitoring tuberculosis treatment outcome: analysis of national surveillance data from a clinical perspective. Thorax. 2008;63(5):440-6. 10.1136/thx.2006.073916 17615085

[30] Gadoev J Asadov D Tillashaykhov M Tayler-Smith K Isaakidis P Dadu A Factors Associated with Unfavorable Treatment Outcomes in New and Previously Treated TB Patients in Uzbekistan: A Five Year Countrywide Study. PLoS One. 2015;10(6):e0128907. 10.1371/journal.pone.0128907 26075615 PMC4467982

[31] de Faria Gomes NM, da Mota Bastos MC, Marins RM, Barbosa AA, Soares LC, de Oliveira Wilken de Abreu AM, et al. Differences between Risk Factors Associated with Tuberculosis Treatment Abandonment and Mortality. Pulm Med. 2015;2015:546106. 10.1155/2015/546106 PMC4639647 26600948

[32] Ducomble T Tolksdorf K Karagiannis I Hauer B Brodhun B Haas W The burden of extrapulmonary and meningitis tuberculosis: an investigation of national surveillance data, Germany, 2002 to 2009. Euro Surveill. 2013;18(12):20436. 23557944

[33] Loytved G Steidle B Benz E Koszczynski W Tuberkulosebekämpfung in Unterfranken 1995 - 2001 - Fallfindung und Behandlungsergebnisse. [Tuberculosis control in lower Franconia 1995 - 2001. Case-finding and treatment outcome]. Pneumologie. 2002;56(6):349-56. 10.1055/s-2002-32171 12063615

[34] Munro SA Lewin SA Smith HJ Engel ME Fretheim A Volmink J Patient adherence to tuberculosis treatment: a systematic review of qualitative research. PLoS Med. 2007;4(7):e238. 10.1371/journal.pmed.0040238 17676945 PMC1925126

[35] Abarca Tomás B Pell C Bueno Cavanillas A Guillén Solvas J Pool R Roura M Tuberculosis in migrant populations. A systematic review of the qualitative literature. PLoS One. 2013;8(12):e82440. 10.1371/journal.pone.0082440 24349284 PMC3857814

[36] Lönnroth K Migliori GB Abubakar I D’Ambrosio L de Vries G Diel R Towards tuberculosis elimination: an action framework for low-incidence countries. Eur Respir J. 2015;45(4):928-52. 25792630 10.1183/09031936.00214014 PMC4391660

